# The histone methyltransferase KMT2D is essential for embryo implantation via regulating precise differentiation of endometrial cells

**DOI:** 10.1038/s41420-024-02134-9

**Published:** 2024-08-08

**Authors:** Ryosuke Kobayashi, Yuki Tajika, Junki Kohmaru, Sumiyo Morita, Takuro Horii, Yoichi Mizukami, Shizu Aikawa, Yasushi Hirota, Izuho Hatada

**Affiliations:** 1 Laboratory of Genome Science, Biosignal Genome Resource Center, Institute for Molecular and Cellular Regulation, Gunma University, Gunma, Japan; 2 Department of Anatomy, Gunma University Graduate School of Medicine, Maebashi, Japan; 3 Gunma Prefectural College of Health Sciences, Maebashi, Japan; 4 Institute of Gene Research, Science Research Center, Yamaguchi University, Yamaguchi, Japan; 5 Department of Obstetrics and Gynecology, Graduate School of Medicine, The University of Tokyo, Tokyo, Japan; 6 Viral Vector Core, Gunma University Initiative for Advanced Research (GIAR), Gunma, Japan

**Keywords:** Gene silencing, Infertility

## Abstract

Embryo implantation failures are a major challenge in reproductive medicine, but the underlying mechanism remains poorly understood. Successful implantation requires dynamic remodeling of the endometrium through integrated proliferation and differentiation of endometrial cells including luminal epithelial, glandular epithelial, and stromal cells. Conversely, their disruption causes infertility. Spatiotemporal control of transcription is required for these processes; however, the underlying epigenetic regulation is largely unknown. In this study, we examined expression data from the human endometrium during implantation and discovered that expression of the histone lysine methyltransferase *KMT2D* was significantly suppressed in patients with recurrent implantation failure. Further study revealed that uterine deletion of *Kmt2d* in mice caused infertility due to implantation failure. Morphological analysis discovered a reduction in the number of uterine glands and aberrant differentiation of the luminal and glandular epithelium into stratified phenotypes in *Kmt2d* knockout uteri. Administration of leukemia inhibitory factor protein, which is expressed in uterine glands and is essential for implantation, did not rescue implantation failure in *Kmt2d* knockout mice, suggesting that infertility was not solely due to uterine gland dysfunction. RNA sequencing analysis revealed that *Kmt2d* knockout uteri displayed suppressed expression of genes involved in ion homeostasis, which may affect the uterine luminal morphology. Our study suggests that KMT2D plays an essential role in facilitating successful embryo implantation by regulating the coordinated differentiation of endometrial cells, providing valuable insights into unexplained implantation failures in women.

## Introduction

Embryo implantation is the first important step for a successful pregnancy in mammals and requires bidirectional communication between the healthy blastocyst and receptive endometrium [1, 2]. The endometrium is a highly dynamic tissue undergoing molecular and cellular changes to transition into a receptive state for embryos during the menstrual cycle in human and the reproductive cycle in rodents. The receptive state is limited to a very short period in early pregnancy, which is called “the window of implantation” [1, 2]. Outside this window, the endometrium becomes refractory to embryos [3, 4]. In human fertility treatment, implantation failure occurs in approximately 60–70% of cases despite the transfer of high-quality embryos [5], presumably due to inadequate endometrial receptivity [6]. Therefore, understanding the regulation of endometrial receptivity is critical to improve the success rates of implantation in fertility treatment.

The endometrium, consisting of luminal and glandular epithelial cells as well as stromal cells, undergoes precise regulation of growth and differentiation by the ovarian steroid hormones estrogen (mainly 17β-estradiol, E2) and progesterone (P4) during establishment of receptivity [1, 2]. In mice, E2 from ovaries promotes epithelial cell proliferation on day 1 of pregnancy (defined as the day on which the vaginal plug is observed), followed by P4 from the newly formed corpora lutea inhibiting E2-induced cell division and promoting epithelial differentiation for embryo implantation [1, 2, 7]. On day 4 of pregnancy, a transient surge of E2 induces secretion of leukemia inhibitory factor (LIF) from uterine glands, initiating embryo attachment via LIF-LIFR-STAT3 signaling [8, 9]. Upon embryo attachment, endometrial stromal cells surrounding the embryo terminally differentiate into decidual cells, supporting subsequent trophoblast invasion and placentation [10]. Imbalance in hormonal signaling disrupts endometrial tissue integrity, leading to implantation failure and infertility [11]. Moreover, improper tissue development in the endometrium, such as uterine gland deficiency or abnormal stratification of epithelium, is known to affect receptivity [12–15].

Dynamic tissue remodeling in the peri-implantation endometrium is manifested by spatiotemporal regulation of gene expression in endometrial cells. Epigenetic machinery, which alters chromatin structure and transcription through DNA/RNA methylation and histone modification, may be the basis of precise transcriptional regulation in the endometrium. Recent studies using mouse models have shown that defects of several epigenetic modifiers in the uterus lead to infertility due to abnormal transcription in endometrial cells [16–24], shedding light on the involvement of epigenetics in endometrial functions. Cell- and tissue-specific gene expression relies on enhancers, which are *cis*-regulatory regions containing transcription factor-binding sites [25, 26]. Enhancers are located far from transcription start sites of genes, and these regions are epigenetically marked by mono-methylation of histone H3 lysine 4 (H3K4me1) [25, 26]. In mammals, enhancer-associated H3K4me1 is mainly catalyzed by the histone lysine methyltransferases KMT2C and KMT2D (also known as MLL3 and MLL4, respectively) with partially redundancy [27]. *KMT2C/D* are important for precise regulation of H3K4me1 levels at enhancer regions and cell-specific transcription [28, 29]. Genetic mutations in *KMT2C/D* are primary causes of neurodevelopmental disorders, such as Kleefstra syndrome 2 and Kabuki syndrome, respectively [30–32]. Moreover, somatic mutations in *KMT2C/D* have been found in many types of cancer including endometrial cancer, indicating these genes are involved in tissue homeostasis and tumorigenesis [33–35]. Although the links between KMT2s and several diseases are becoming clear, the importance of KMT2C/D for tissue development and homeostasis of reproductive organs is largely unknown.

Here, we found that *KMT2D* expression was decreased in patients with recurrent implantation failure (RIF). To explore the role of *KMT2D* in the endometrium, we deleted *Kmt2d* in murine uterus and showed that *Kmt2d* is indispensable for successful embryo implantation. *Kmt2d* deficiency induced morphological abnormalities in the uterus, including a reduced uterine gland and abnormal epithelium differentiation, which impaired endometrial receptivity. Our findings not only reveal that *KMT2D* plays a critical role in regulation of uterine morphology and function in mice, but also suggest that *KMT2D* suppression may influence unexplained implantation failures in human patients.

## Results

### Expression of *KMT2* genes is decreased in the endometrium of patients with RIF

**Fig. 1:**
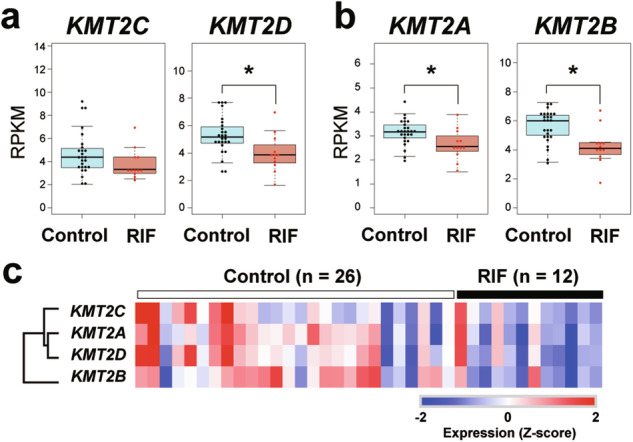
Expression of *KMT2* genes is suppressed in the endometrium of patients with RIF. RPKM values of *KMT2C/D* (**a**) and *KMT2A/B* (**b**) in the endometrium of control (n = 26) and RIF (n = 12) patients. *p < 0.05, two-tailed Mann–Whitney U test. **c** Heatmap of *KMT2* expression in the human endometrium.

A prior study using RNA-seq data from the human endometrium during implantation found that downregulation of epigenetic factors like *EZH1/2* and *EEH* in patients with RIF [20]. Using the same dataset, we further examined the expression of genes involved in enhancer activity, *KMT2*C and *KMT2D*, in RIF patients. Comparison of the reads per kilobase of transcript per million mapped reads (RPKM) values of *KMT2* genes between fertile control and RIF patients revealed that *KMT2D* expression was significantly decreased in RIF patients, but *KMT2C* expression was not (Fig. 1a). Moreover, *KMT2A* and *KMT2B*, which code enzymes for di- and tri-methylation of H3K4 [27], were also downregulated in RIF patients (Fig. 1b). A heatmap of *KMT2* gene expression showed that samples with low *KMT2D* expression also had low expression of other *KMT2* genes (Fig. 1c). Indeed, the RPKM value of *KMT2D* correlated well with other *KMT2* genes in human endometrium (R^2^ = 0.7802 *vs. KMT2A*, R^2^ = 0.5576 *vs*. *KMT2B*, and R^2^ = 0.7774 *vs*. *KMT2C*). Our analysis demonstrated that expression of *KMT2* genes was concomitantly decreased in the endometrium of RIF patients.

### Uterine deletion of *Kmt2d* in mice caused infertility due to implantation failure

**Fig. 2:**
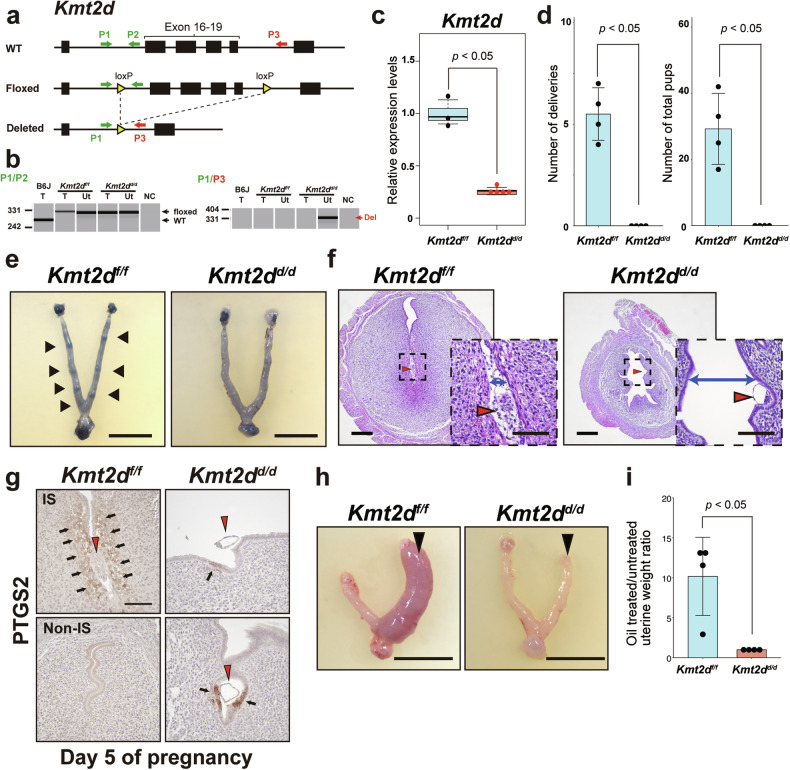
Deletion of *Kmt2d* in the uterus leads to infertility due to implantation failure. **a** The strategy to conditionally delete the *Kmt2d* gene. P1–3 indicate the locations of primers used for genotyping. **b** Genotyping PCR results showing *Kmt2d* was deleted in the uterus. T, tail DNA; Ut, uterus DNA; NC, negative control (water). **c** Decreased expression of *Kmt2d* gene in *Kmt2d*^*d/d*^ uteri was confirmed by RT-qPCR analysis. **d** The results of a 6-month breeding assay. The numbers of deliveries (left) and total pups (right) were lower for *Kmt2d*^*d/d*^ mice than for *Kmt2d*^*f/f*^ mice. n = 4 per group. *P*-values were calculated with the two-tailed Mann–Whitney U test. **e** Representative images of the gross anatomy of uteri on day 5 of pregnancy in control (left) and *Kmt2d*^*d/d*^ (right) mice. Implantation sites were visualized by injected blue dye in *Kmt2d*^*f/f*^ uteri, but not in *Kmt2d*^*d/d*^ uteri. Scale bar = 10 mm. **f** Cross-sections of implantation sites on day 5 of pregnancy. Insets show embryos (red arrowheads) in the uterine cavity. The uterine lumen in *Kmt2d*^*f/f*^ mice was tightly closed to contact with embryos, but *Kmt2d*^*d/d*^ uteri displayed a space within the lumen due to abnormal closure. Luminal spaces are indicated by blue double-headed arrows. Scale bars indicate 250 μm (low magnification images) and 100 μm (insets). **g** Immunohistochemistry of PTGS2 in day 5 uteri of *Kmt2d*^*f/f*^ and *Kmt2d*^*d/d*^ mice. In the *Kmt2d*^*f/f*^ uterus, PTGS2 expression (black arrows) was observed in stroma around the implantation site (IS) but was absent in the non-IS. PTGS2 expression in the *Kmt2d*^*d/d*^ uterus was mainly detected in the epithelium around the embryo (the embryo is indicated by a red arrowhead). Scale bar = 100 μm. **h** Representative images showing the gross anatomy of the oil-injected (arrowhead, right horn) and untreated (left horn) uterine horns of *Kmt2d*^*f/f*^ and *Kmt2d*^*d/d*^ females at 3 days after oil injection. Scale bar = 10 mm. **i** The ratio of the weight of the oil-injected uterine horn (n = 4) to that of the untreated uterine horn (n = 4). The *P*-value was calculated using the two-tailed Mann-Whitney U test.

*KMT2D* downregulation in the endometrium of RIF patients suggested a critical role of *KMT2D* during implantation. To investigate the roles of *KMT2D* in the uterus, we generated mice with conditional knockout of *Kmt2d* in the uterus by crossing *Kmt2d*-floxed (*Kmt2d*^*f/f*^) mice with *Pgr*^*Cre*^ mice [24], which expressed Cre recombinase in the uterus. Uterine deletion of *Kmt2d* (*Pgr*^*Cre*^*, Kmt2d*^*f/f*^; hereafter described as *Kmt2d*^*d/d*^) was confirmed in DNA (Fig. 2a, b), and mRNA levels (Fig. 2c). Female fertility was assessed by paring *Kmt2d*^*d/d*^ or *Kmt2d*^*f/f*^ female (as control) mice with wild-type male for 6 months, and litters and pups were tracked. While all control mice produced pups, *Kmt2d*^*d/d*^ females did not become pregnant and give birth (Fig. 2d). Next, we analyzed whether *Kmt2d* ablation affected implantation. By the evening on day 5 of pregnancy, *Kmt2d*^*f/f*^ uteri showed apparent implantation sites, but no implantation sites were observed in *Kmt2d*^*d/d*^ uteri (Fig. 2e). Histological analysis indicated that embryos were implanted in the endometrium, tightly contacted with closed lumen, and surrounded by decidua in *Kmt2d*^*f/f*^ uteri (Fig. 2f, left). Conversely, in *Kmt2d*^*d/d*^ uteri, embryos lay on the uterine luminal epithelium with the endometrial lumen remaining open (Fig. 2f, right), indicating implantation was compromised. Expression of PTGS2, a key enzyme for prostaglandin synthesis (also known as COX2), was detected in the stroma surrounding the embryo in control uteri on day 5 (Fig. 2g), indicating successful embryo attachment [36, 37]. By contrast, PTGS2 expression was mainly detected on the epithelium surrounding the embryo in *Kmt2d*^*d/d*^ uteri (Fig. 2g). This resembles the patterns observed in *Lif*^*−/−*^ and *Msx1/2*^*d/d*^ mice, which are infertile due to implantation failure [38, 39], suggesting impaired implantation in *Kmt2d*^*d/d*^ uteri.

*Kmt2d*^*d/d*^ uteri showed no signs of decidua on day 5; therefore, we next examined whether *Kmt2d* ablation impacts decidualization in the uterus using an artificial model [40]. Sesame oil was injected into uteri of pseudopregnant mice and decidualization was examined 2 days later. *Kmt2d*^*f/f*^ uteri exhibited a clear decidual response (Fig. 2h, left). However, *Kmt2d*^*d/d*^ uteri did not form decidua after oil injection (Fig. 2h, right). The stimulated uterine horn of *Kmt2d*^*d/d*^ mice weighed less than that of *Kmt2d*^*f/f*^ mice (Fig. 2i), indicating impaired decidualization in *Kmt2d*^*d/d*^ uteri.

The phenotypes of ovaries in *Kmt2d*^*d/d*^ mice were also examined, but we did not find any histological abnormality compared with ovaries of *Kmt2d*^*f/f*^ mice (Fig. S1a). Serum levels of E2 and P4 on day 4 of pregnancy were also comparable (Fig. S1b). Thus, we concluded that infertility of these mice was due to abnormal uterine functions.

*KMT2C* is a paralog of *KMT2D* and they share several functional redundancies [27]; therefore, we examined the role of *KMT2C* in the uterus using *Kmt2c* knockout females (*Pgr*^*Cre*^*, Kmt2c*^*f/f*^; hereafter described as *Kmt2c*^*d/d*^, Fig. S2a, b). *Kmt2c*^*d/d*^ females had normal pregnancies from embryo implantation (Fig. S2c) to parturition (Fig. S2d). *Kmt2c* deletion did not have apparent effects on female fertility; therefore, subsequent analyses focused on *Kmt2d*.

### *Kmt2d* ablation alters responsive signaling in the uterine epithelium

**Fig. 3:**
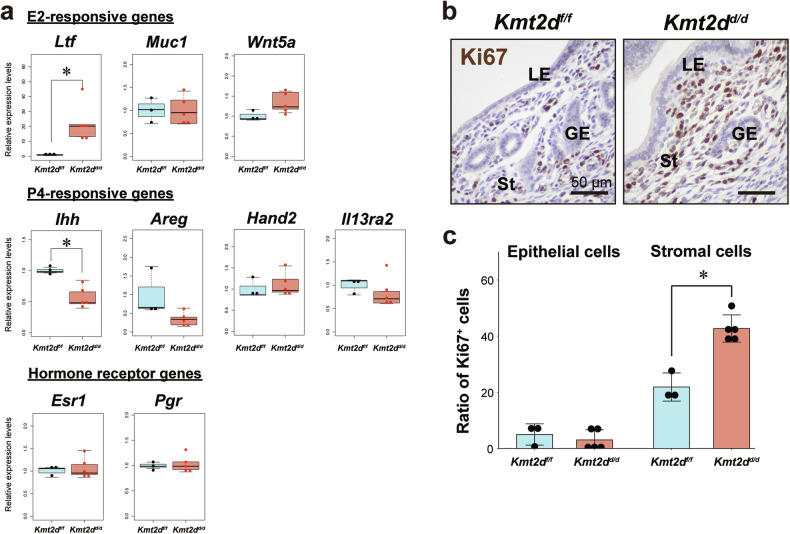
Molecular indexes of uterine receptivity are compromised in *Kmt2d*^*d/d*^ uteri. **a** Expression analysis of E2- and P4-responsive genes by RT-qPCR. n = 3 and 5 in *Kmt2d*^*f/f*^ and *Kmt2d*^*d/d*^, respectively. *p < 0.05, two-tailed Mann–Whitney U test. **b** Cell proliferation in uteri on day 4 of pregnancy determined by immunohistochemistry of the cell proliferation marker Ki67. LE luminal epithelium, GE glandular epithelium, St stroma. Scale bar = 50 μm. **c** Percentages of Ki67-positive luminal epithelial cells (left) and stromal cells (right). *p < 0.05, two-tailed Mann–Whitney U test.

Next, we examined whether *Kmt2d* ablation affected the molecular status of endometrial receptivity. The implantation window in the mouse uterus occurs on day 4 of pregnancy, during which the endometrium transitions from an E2-dominant state to a P4-responsive state [1, 2]. Dysregulation of hormonal signaling in the endometrium affected receptivity [11]; therefore, we analyzed E2- and P4-responsive gene expression in *Kmt2d*^*d/d*^ uteri on day 4 of pregnancy. Among E2-responsive genes, whose expression is normally suppressed on day 4 of murine pregnancy, *Ltf* was significantly upregulated in *Kmt2d*^*d/d*^ uteri, while *Muc1* and *Wnt5a* expressions were comparable in *Kmt2d*^*d/d*^ and *Kmt2d*^*f/f*^ uteri (Fig. 3a). *Ihh*, an epithelial P4-targeted gene, was markedly downregulated in *Kmt2d*^*d/d*^ uteri (Fig. 3a). Another epithelial P4-targeted gene, *Areg*, was not altered in *Kmt2d*^*d/d*^ uteri. Expression of stromal P4-responsive genes such as *Hand2* and *Il13ra2* remained unchanged (Fig. 3a). Expression of hormone receptors including *Esr1* and *Pgr* was not altered in *Kmt2d*^*d/d*^ uteri (Fig. 3a). These data suggested that *Kmt2d* ablation altered hormone-responsive signaling, especially in the uterine epithelium.

### Epithelial proliferation during implantation is inhibited in *Kmt2d*^*d/d*^ uteri

During implantation in mouse, increased P4 signaling terminates proliferation of endometrial epithelial cells and increases the proliferation activity of stromal cells [4]. RT-qPCR analysis showed that P4 signaling was inhibited in the *Kmt2d*^*d/d*^ uterine epithelium on day 4 of pregnancy (Fig. 3a); therefore, we expected epithelial proliferation to be aberrantly upregulated in *Kmt2d*^*d/d*^ uteri. Immunostaining of Ki67, a marker of proliferating cells, was performed to identify proliferation status. In *Kmt2d*^*f/f*^ uteri, Ki67-positive cells were rarely observed in the epithelium as previously reported (Fig. 3b, left). Unexpectedly, epithelial proliferation was also inhibited in *Kmt2d*^*d/d*^ uteri (Fig. 3b, right, and Fig. 3c). On the other hand, the Ki67-positive proliferating cells in the stroma were increased in *Kmt2d*^*d/d*^ uteri (Fig. 3c). Epithelial proliferation was apparent in estrus and on day 1 of pregnancy in both *Kmt2d*^*f/f*^ and *Kmt2d*^*d/d*^ uteri (Fig. S3). These results suggested that inhibition of epithelial proliferation during implantation was correctly controlled in *Kmt2d*^*d/d*^ uteri despite abnormal ovarian hormonal signaling in *Kmt2d*^*d/d*^ endometrium.

### *Kmt2d* is crucial for precise development of uterine glands

**Fig. 4:**
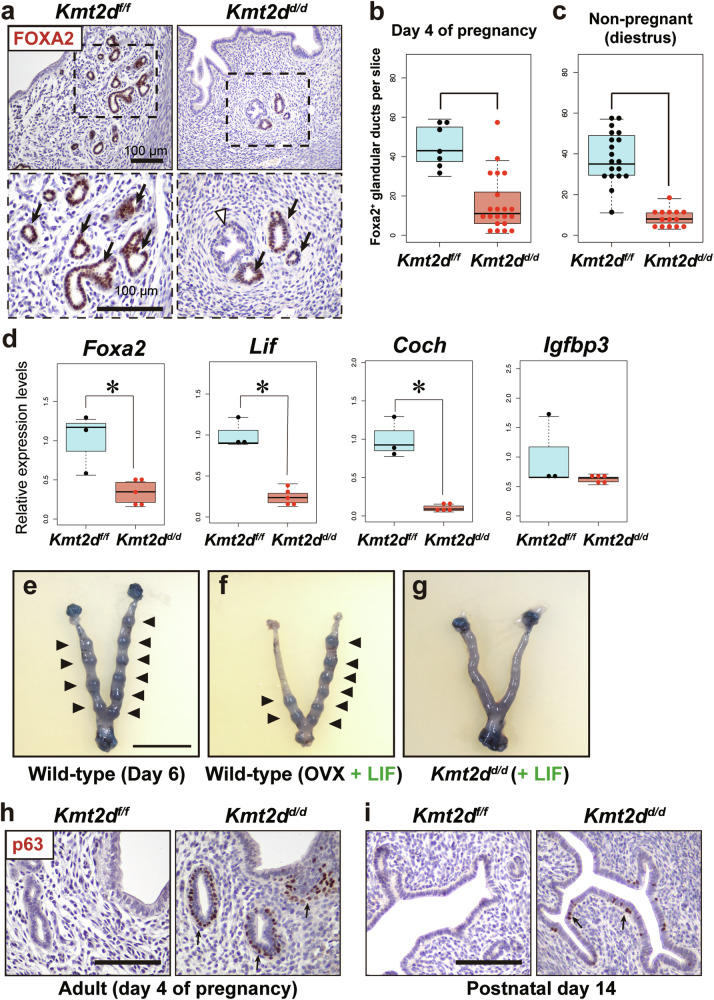
Ablation of *Kmt2d* in the uterus impairs gland development and normal epithelial differentiation. **a** Immunohistochemistry for FOXA2, a marker of endometrial glandular epithelial cells, in the uterus on day 4 of pregnancy. The number of FOXA2-positive glandular ducts per cross-section of the uterus on day 4 of pregnancy (**b**) and of the non-pregnant uterus in diestrus **c**. At least three females were analyzed per group. **d** Expression of genes related to LIF signaling in day 4 uteri examined by RT-qPCR. n = 3 and 5 in *Kmt2d*^*f/f*^ and *Kmt2d*^*d/d*^, respectively. *p < 0.05, two-tailed Mann–Whitney U test. **e**–**g** Rescue experiment of implantation failure by LIF supplementation. **e** Black arrowheads indicate implantation sites on day 6 of a normal pregnancy in a wild-type C57BL/6J female mouse. Scale = 10 mm. **f** Treatment with recombinant LIF induced implantation in a delayed implantation model using wild-type female mice. **g** Implantation sites were not observed in *Kmt2d*^*d/d*^ uteri at 2 days after supplementation of recombinant LIF on day 4 of pregnancy. Histology and immunolocalization of p63, a marker of basal cells in the stratified epithelium, in adult (**h**, day 4 of pregnancy) and developing (**i**, postnatal day 14) uteri. p63-positive cells (black arrows) were present in *Kmt2d*^*d/d*^ uteri, but not in *Kmt2d*^*f/f*^ uteri. Scale bar = 100 μm.

During histological observations, we found that *Kmt2d*^*d/d*^ uteri had fewer uterine glands than control uteri. Immunohistochemistry for FOXA2, a marker of uterine glandular epithelial cells, confirmed that the FOXA2-positive uterine glands were reduced in *Kmt2d*^*d/d*^ uteri during implantation (Fig. 4a, b) and in non-pregnancy (Fig. 4c). Uteri were visualized in 3D by correlative microscopy and block-face imaging to confirm these results of 2D histological observation (Fig. S4, and Movies S1, S2) [41]. The 3D morphological data confirmed the reduction of uterine glands at the whole-tissue level. Notably, a reduction in FOXA2-positive gland cells was already observed in the developing uterus (postnatal day 14, Fig. S5). These observations revealed that *Kmt2d* is essential for precise development of uterine glands.

### Recombinant LIF does not rescue implantation failure in *Kmt2d*^*d/d*^ females

The primary function of uterine glands during implantation is to secrete LIF, an essential cytokine for initiation of implantation [8]. To examine whether reduction of glands in *Kmt2d*^*d/d*^ uteri leads to insufficiency of LIF, we assessed the expressions of *Lif* and its downstream genes in the uterus during implantation. The diminished *Foxa2* expression in *Kmt2d*^*d/d*^ uteri confirmed reduced gland number in *Kmt2d*^*d/d*^ uteri (Fig. 4d). *Lif* and downstream *Coch* were significantly downregulated in *Kmt2d*^*d/d*^ uteri, although expression of another downstream gene, *Igfbp3*, was comparable in *Kmt2d*^*f/f*^ and *Kmt2d*^*d/d*^ uteri (Fig. 4d). These findings indicated that LIF signaling was compromised in *Kmt2d*^*d/d*^ uteri during implantation.

A previous study reported that administration of recombinant LIF rescues implantation failure in gland-deficient mice [42]. We examined whether the implantation failure in *Kmt2d*^*d/d*^ mice was also rescued by LIF administration. In normal pregnancies of wild-type females, implantation sites were apparent on day 6 of pregnancy (Fig. 4e). Administration of recombinant LIF protein effectively induced implantation in delayed implantation model mice, confirming its biological activity (Fig. 4f). However, LIF administration on day 4 did not induce implantation in *Kmt2d*^*d/d*^ females (Fig. 4g, n = 3). These findings indicated that implantation failure in *Kmt2d*^*d/d*^ mice was not solely due to dysfunction of uterine glands.

### A stratified epithelium appears in *Kmt2d*^*d/d*^ uteri

We also identified that some glandular structures exhibited a stratified morphology in *Kmt2d*^*d/d*^ uteri (Fig. 4a, white arrowhead). Immunostaining for p63, a marker of stratified basal cells, confirmed that p63-positive basal cells were present in the luminal and glandular epithelium in adult *Kmt2d*^*d/d*^ uteri, but were completely absent in control uteri (Fig. 4h). p63-positive cells had already appeared in the developing uterus on postnatal day 14 in *Kmt2d*^*d/d*^ females (Fig. 4i). These results suggested that *Kmt2d* plays a pivotal role in the development programs of the endometrial epithelium.

### Genes related to ion homeostasis are downregulated in *Kmt2d*^*d/d*^ uteri

**Fig. 5:**
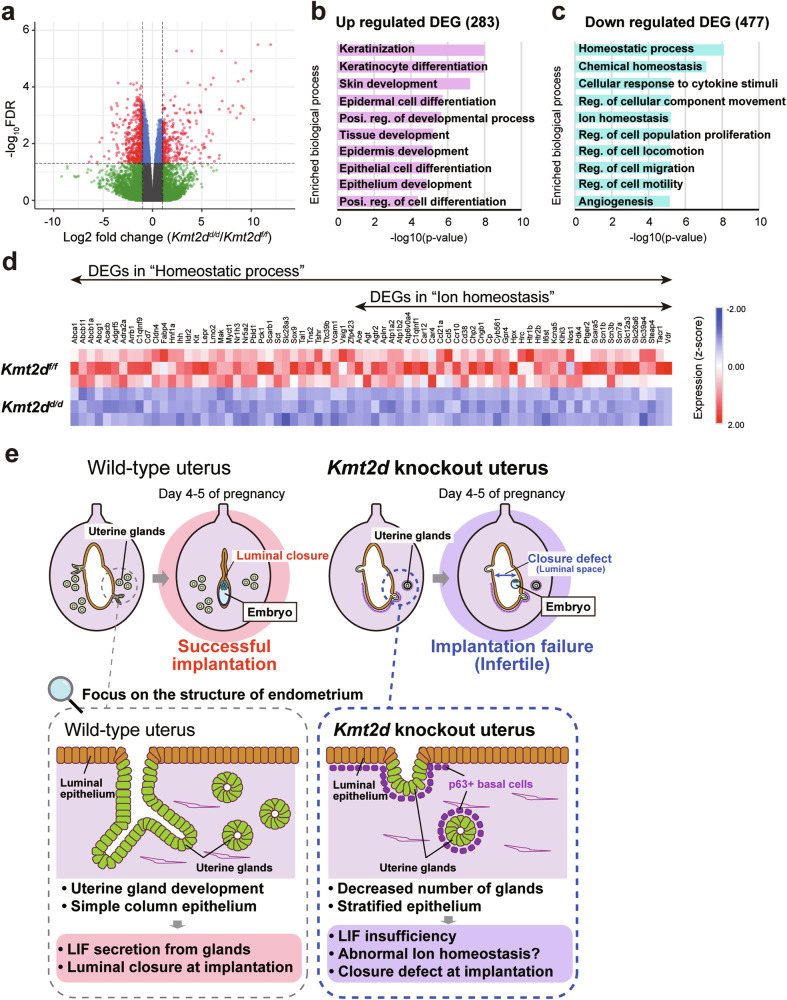
Molecular characterization of *Kmt2d*^*d/d*^ uteri. **a** A volcano plot showing gene expression profiles determined by RNA-seq. DEGs (q < 0.05 and fold change >2) between *Kmt2d*^*f/f*^ and *Kmt2d*^*d/d*^ uteri on day 4 of pregnancy are illustrated in red. Enriched biological processes in upregulated (**b**) and downregulated (**c**) DEGs. **d** Heatmap of expression of DEGs related to the term “Homeostatic process” from RNA-seq data. **e** Graphical summary of the findings about implantation failure in the *Kmt2d*^*d/d*^ mouse model.

To further explore the cause of infertility in *Kmt2d*^*d/d*^ mice, we conducted RNA-seq analysis on day 4 of pregnancy. We identified that 283 and 477 genes were upregulated and downregulated in *Kmt2d*^*d/d*^ uteri, respectively (Fig. 5a and Dataset S1). Gene ontology analysis revealed that upregulated genes in *Kmt2d*^*d/d*^ uteri were highly enriched in “Keratinocyte differentiation” (Fig. 5b). Keratinocytes are the major cell type in the stratified skin epidermis, consistent with our observation that stratified epithelium appeared in *Kmt2d*^*d/d*^ uteri. Downregulated genes were enriched in “Homeostasis” and “Cell migration” (Fig. 5c). In “Homeostatic process”, 74 genes were downregulated, including 39 genes related to “Ion homeostasis”, such as those encoding sodium ion (*Scn1b*, *Scn3b*, and *Scn7a*) and potassium ion (*Kcna5*) channels (Fig. 5d). Given that regulation of ion homeostasis in the uterine lumen is essential for precise uterine function [43–45], our data suggested that an imbalance of ion homeostasis underlies the infertility of *Kmt2d*^*d/d*^ females.

Given the resemblance of the hypo-development of uterine glands in *Kmt2d*^*d/d*^ mice to *Foxa2*^*d/d*^ mice, we compared the transcriptomes of these two strains using a published RNA-seq dataset (GSE113065). Downregulated genes in *Foxa2*^*d/d*^ uteri on day 4 also exhibited lower expression levels in *Kmt2d*^*d/d*^ uteri (Fig. S6). This observation suggested that KMT2D and FOXA2 regulate common gene sets in the murine endometrium.

Our mouse model reveals the critical roles of KMT2D in precise differentiation of endometrial cells and successful implantation. Ablation of *Kmt2d* in murine uterus resulted in infertility due to implantation failure, accompanied by uterine gland hypo-development and abnormal stratified differentiation of the uterine epithelium (Fig. 5e). *Kmt2d* ablation also altered transcriptome programs in the endometrium, as exemplified by downregulation of genes related to ion homeostasis. Thus, we propose that KMT2D regulates multiple layers of functions for successful implantation in the endometrium, and *Kmt2d* deficiency has serious consequences for female fertility.

## Discussion

The KMT2 family consists of highly conserved proteins, which has a catalytic SET domain that methylates lysine residues of histone H3 [28]. Among them, KMT2C and KMT2D share a common ancestral homolog, *Drosophila* Trithorax-related (Trr), which regulates enhancer activity by catalyzing mono-methylation of H3K4 to the target regions [46]. KMT2C/D have highly similar protein structures and therefore share some level of redundant functions [27, 29]. However, systemic knockout of *Kmt2c* or *Kmt2d* in mice results in embryonic lethality at different stages [47], suggesting that the importance of *KMT2C/D* for mammalian development is not equivalent and varies according to the stage and tissue. In this study, we found that expression of *KMT2D*, but not *KMT2C*, was downregulated in the endometrium of patients with RIF (Fig. 1). Consistent with expression data from patient samples, our mouse model revealed that deletion of *Kmt2d* in uterus led to female infertility due to implantation failure (Fig. 2), but knockout of *Kmt2c* did not. Thus, *Kmt2d* appears to be more important than *Kmt2c* for uterine development and function. Somatic mutations of *KMT2D* are found more frequently than *KMT2C* mutations in human endometrial cancer [35], indicating the tissue-specific importance of *KMT2* genes. *KMT2D* mutations have also been observed in Kabuki syndrome patients. However, these patients (both male and female) appear to be fertile [48], suggesting that haploinsufficiency of *KMT2D* does not significantly affect uterine function. We found that other *KMT2* genes in addition to *KMT2D* were simultaneously downregulated in the endometrium of RIF patients; therefore, suppression of multiple *KMT2* genes may distort epigenetic regulation in the endometrium, resulting in implantation failure. Indeed, it was reported that KMT2B can compensate for the enhancer activity regulated by KMT2C/KMT2D [49]. A future study needs to identify the cause of simultaneous suppression of *KMT2* genes in the endometrium of RIF patients.

Epithelial proliferation in the endometrium is inhibited during murine implantation, which is a hallmark of a receptive uterus, whereas stromal cells increase their growth [4]. These states are both achieved by increased P4-PGR signaling in the endometrium at the time of implantation [50]. Intriguingly, the proliferation of stromal cells in *Kmt2d*^*d/d*^ endometrium was more pronounced than in control mice (Fig. 3c), but no abnormal enhancement of P4-PGR signaling was observed in the *Kmt2d*^*d/d*^ uteri (Fig. 3a, and Fig. S1). Thus, the increased proliferation in stromal cells may not be due to dependent on P4-PGR signaling, but rather other causes. Although it is not clear whether the increased stromal proliferation itself is a direct cause of implantation failure in *Kmt2d*^*d/d*^ mice, we observed evidence indicating abnormalities in the characteristics of stromal cells in the knockout mice. PTGS2 (COX2) expression typically propagates from the epithelium to the stroma during implantation, but this propagation is scarcely observed in the knockout uterus (Fig. 2g). Additionally, stromal cells differentiate into decidual cells that support fetal growth, but there is no decidualization in the uteri of *Kmt2d*^*d/d*^ mice (Fig. 2f and g). These abnormalities in uterine stromal cells may partially contribute to the implantation defect in *Kmt2d*^*d/d*^ mice.

The prominent phenotype of *Kmt2d*^*d/d*^ uteri was endometrial gland reduction. Uterine gland development (i.e., adenogenesis) begins after birth in mice and is completed by 3 weeks of age [51]. Foxa2 is the master regulator of uterine adenogenesis because mice lacking *Foxa2* in the uterus shortly after birth suffer from uterine gland aplasia [14]. FOX family transcription factors, including FOXA2, are pioneer factors that open up compacted chromatin by preferentially binding to heterochromatin regions during cell differentiation, contributing to formation of cell-specific enhancers and subsequent gene expression in post-differentiated cells [52, 53]. In the colon, binding of FOXD2 to compacted chromatin facilitates recruitment of KMT2D and rewiring enhancer through deposition of H3K4me1, which functions as a tumor suppressor [54]. Although *Foxa2* expression was decreased in *Kmt2d*^*d/d*^ uteri, this may reflect the reduced number of uterine glands. Instead, loss of *Kmt2d* may disrupt formation of FOXA2-regulated enhancers, leading to defective development of uterine glands in *Kmt2d*^*d/d*^ females. Our observation that downregulated genes in *Foxa2*^*d/d*^ uteri also exhibited lower expression levels in *Kmt2d*^*d/d*^ uteri supports the idea that *Kmt2d* and *Foxa2* operate in the same regulatory pathway for glandular development in the murine endometrium (Fig. S6).

Implantation failure in mice lacking uterine glands due to *Foxa2* deletion is rescued by LIF supplementation [42], demonstrating that the most critical role of uterine glands during murine implantation is to secrete LIF. Although *Kmt2d*^*d/d*^ mice also exhibited fewer uterine glands, LIF administration alone did not rescue implantation failure (Fig. 4g), suggesting that endometrial receptivity in *Kmt2d*^*d/d*^ mice is disrupted at a stage before LIF action. While we failed to detect abnormal receptivity in *Kmt2d*^*d/d*^ uteri in terms of inhibition of epithelial proliferation, *Kmt2d*^*d/d*^ uteri may lack other factors necessary for embryo implantation, such as protein secretion or expression of adhesion molecules in the luminal epithelium. Further experiments are required to elucidate why LIF administration does not rescue implantation failure in *Kmt2d*^*d/d*^ mice.

Stratification of the uterine epithelium was apparent in *Kmt2d*^*d/d*^ uteri. During development of female reproductive ducts, the Müllerian duct epithelium in the fetus becomes a monolayer of uterine epithelial cells or stratified vaginal epithelial cells dependent on paracrine factors released by stromal cells, such as bone morphogenetic proteins (BMPs) and fibroblast growth factors (FGFs) [55, 56]. The stratified epithelium is completely absent in the normal uterus in rodents and human, but appears in pathological conditions such as endometrial cancer [57, 58]. Abnormal stratification is also observed in uteri of several genetically modified mice, such as strains with knockout of *Fgfr2* and *Wnt4* [15, 59]. In *Kmt2d*^*d/d*^ uteri, expression of several genes related to the BMP, FGF, and WNT pathways was abnormal compared with control (Fig. S7). Disrupted paracrine signaling likely alters the epithelial differentiation program, resulting in abnormal stratification of the uterine epithelium in *Kmt2d*^*d/d*^ uteri. In the epithelium, a morphological difference (i.e., single layer or stratified) reflects functional differences; thus, several epithelial functions, including protein secretion and absorption, are impaired in *Kmt2d*^*d/d*^ uteri, resulting in abnormal receptivity.

As described above, *Kmt2d*^*d/d*^ females exhibited significant morphological abnormalities in the uterus. Since the public human data used for the RNA-seq analysis excluded patients with known uterine abnormalities [20], it is likely that no known uterine morphological abnormalities occur in the uteri of patients with RIF in which KMT2D suppression is present. Possible explanations for the discrepancy between human patients and the mouse model regarding uterine morphological abnormalities include the following: (1) In the patient’s uterus, unlike in the knockout model, KMT2D expression was not completely abolished, resulting in functional but not morphological abnormalities. (2) In the mouse model, KMT2D knockout begins soon after birth, leading to uterine developmental abnormalities (see Fig. 4i, and Fig. S5), whereas in the human patient’s uterus, the reduction in KMT2D expression may be an acquired event (i.e., occurring in adulthood) and not accompanied by uterine morphological abnormalities. Future work is needed to generate and validate a model in which KMT2D knockout occurs in the uterus after sexual maturation.

Transcriptome analysis of peri-implantation uteri revealed that *Kmt2d* ablation decreased expression of genes related to homeostasis, especially ion homeostasis. Absorption and secretion of ions such as sodium and chloride ions in the luminal epithelium regulate fluid volumes in the endometrial lumen through osmotic pressure [43–45]. Mechanistically, active sodium ion uptake drives fluid absorption from the lumen into the blood, while chloride ion secretion drives fluid movement from the blood into the lumen, concomitant with sodium ion secretion [43, 45]. At the time of implantation, the uterine lumen must close tightly in contact with the embryo (Fig. 2f, left panel, and Fig. 5e), a process that is supposedly mediated by absorption of luminal fluid in the uterine epithelium [60]. By contrast, the lumen did not close at the time of implantation in *Kmt2d*^*d/d*^ uteri (Fig. 2f, right panel, and Fig. 5e). Improper control of ion concentrations in the uterine lumen may result in retention of uterine luminal fluid, causing failure of luminal closure in *Kmt2d*^*d/d*^ uteri. Furthermore, suppression of epithelial sodium ion channels in the murine uterus triggers implantation failure via inhibition of prostaglandin E2 release and decidualization defects [44]. Thus, ion homeostasis in the uterus is involved in protection of successful implantation in multiple respects.

The DEGs involved in “ion homeostasis”, which were reduced in *Kmt2d*^*d/d*^ mice, also included genes encoding voltage-gated sodium channels, such as *Scn1b*, *Scn3b*, and *Scn7a* (Fig. 5d), which are primarily expressed in muscle and nerve cells. Recent studies have demonstrated that functional voltage-gated sodium channels are expressed in non-excitable cells, including mammary epithelial cells [61], retinal pigment cells [62], and macrophages [63]. These genes play roles in cell adhesion and phagocytosis [61–63]. Therefore, the voltage-gated sodium channel genes downregulated in *Kmt2d*^*d/d*^ mice may also be involved in the regulation of embryo implantation via ion homeostasis through non-canonical functions. Another possible explanation is that since RNA-seq is performed using RNA extracted from the whole uterus, uterine smooth muscle contraction or dysregulation of neural activity, which is sparsely present in the uterus, may influence implantation failure.

In summary, we revealed that KMT2D plays a critical role in embryo implantation through precise regulation of uterine development and homeostasis in mice. Recent studies using animal models revealed that other histone modifiers such as EZH2, CFP1, and HDAC3 also have indispensable roles in maintaining uterine transcriptome networks to prepare the endometrium for embryo implantation [16–18, 20, 21, 64, 65]. Our results enhance understanding of epigenetic regulation in the endometrium and are expected to pave the way for future advancements in the diagnosis and treatment of unexplained implantation defects.

## Materials and methods

### Animal experiments

C57BL/6J and ICR mice were obtained from Charles River Japan (Yokohama, Japan). B6D2F1 mice were purchased from CLEA Japan (Kawasaki, Japan). All mice were housed in a specific pathogen-free facility with a 12 h dark/light cycle, and were chosen randomly without blinding. All animal studies were approved by the Animal Care and Experimentation Committee of Gunma University (approved No. 23-048) and were carried out in accordance with approved guidelines.

For implantation analysis, females were crossed with a wild-type male in the same cage. Day 1 of pregnancy was defined as the day on which the vaginal plug was identified. Implantation sites were visualized by intravenous injection of 2% Evans blue dye dissolved in phosphate-buffered saline (PBS) at 17:00 on day 5 of pregnancy. When uterine tissues were harvested on day 4 of pregnancy, blastocysts were recovered by flushing the uterine horns with PBS to evaluate embryo development prior to implantation. Uteri from which blastocysts were not recovered were not used in subsequent experiments. For the breeding assay, mature females (>7 weeks old) were mated with wild-type C57BL/6J males over 6 months. The numbers of litters and pups were recorded.

#### Generation of uterine-specific *Kmt2c/Kmt2d* conditional knockout mice

*Kmt2c-* and *Kmt2d*-floxed mice (*Kmt2c*^*f/f*^ and *Kmt2d*^*f/f*^) were generated using an electroporation method as previously reported [66]. Single-stranded donor oligodeoxynucleotides (ssODNs) with 5′- and 3′-homology arms corresponding to target sequences, flanking *loxP*, and containing a restriction site were designed (Fig. 1a and SI Appendix, Fig. S1a). Opti-MEM I (Life Technologies, Carlsbad, CA) containing pre-annealed crRNA/tracrRNA (3 μM), recombinant Cas9 protein (100 ng μl^−1^; GeneArt Platinum Cas9 nuclease; Thermo Fisher Scientific, Waltham, MA), and ssODN (400 ng μl^−1^; Ultramer; Integrated DNA Technologies, Coralville, IA) was used as the electroporation medium. A left loxP site was introduced into the intron between exons 8 and 9 of *Kmt2c* for *Kmt2c*^*f/f*^ or exons 15 and 16 of *Kmt2d* for *Kmt2d*^*f/f*^ by electroporation using C57BL/6J-derived zygotes, and then edited embryos were transferred to the oviduct of a pseudopregnant ICR female mouse to obtain males with one loxP site in the left region. Next, a right loxP site was inserted into the intron between exons 13 and 14 of *Kmt2c* or exons 19 and 20 of *Kmt2d* by genome editing using left loxP site-containing male-derived zygotes to obtain *Kmt2c-* and *Kmt2d*-floxed mice. Sequence information of the crRNAs and ssODNs is provided in Table S1. Uterine-specific *Kmt2c* and *Kmt2d* knockout mice (*Kmt2c*^*d/d*^ and *Kmt2d*^*d/d*^) were established by several crossings of the *Pgr*^*Cre*^ strain [24] with floxed mice. Floxed females in the same litters as knockout animals were used as controls unless otherwise noted. The primers used for PCR analysis to assess uterine-specific deletion of *Kmt2c* and *Kmt2d* are listed in Table S2.

#### Histology

Collected murine tissues were fixed in PBS (pH 7.4) containing 4% paraformaldehyde and embedded in paraffin blocks. For histological analysis, sections (3 μm) were stained with hematoxylin and eosin (H&E). For immunohistochemistry, the sections were deparaffinized and incubated in 10 mM sodium citrate buffer (pH 6.0) at 121 °C for 5 min for antigen retrieval. The sections were further incubated with 3% hydrogen peroxide diluted with methanol for 15 min. After incubation with blocking buffer (PBS containing 10% goat serum, 1% bovine serum albumin, and 0.05% Tween 20), the sections were reacted with primary antibodies against PTGS2 (66351-1-Ig; Proteintech, Rosemont, IL), Ki67 (ab15580; Abcam, Cambridge, UK), FOXA2 (ab108422, Abcam), and p63 (ab124762, Abcam). The sections were then reacted with Histofine Simple Stain MAX-PO (Nichirei, Tokyo, Japan). Signals were developed with 3-3′-diaminobenzidine, followed by counterstaining with hematoxylin.

#### Artificial decidualization test

The artificial decidualization test was performed by intra-luminally injecting 0.02 mL of sesame oil into pseudo-pregnant female mice at 12:00 on day 4 of pseudo-pregnancy. These mice were generated by mating with a vasectomized male as previously described [40]. The uterine horns were dissected 2 days after oil injection and weighed.

#### Reverse transcription-quantitative PCR (RT-qPCR) analysis

Uteri on day 4 of pregnancy were harvested and kept at −80 °C until RNA extraction. Total RNA was isolated from uterine tissues using TRIzol reagent (Invitrogen, Carlsbad, CA). Following DNase I treatment and RNA purification using a FastGene RNA Premium Kit (Nippon Genetics, Tokyo, Japan), cDNA was synthesized using SuperScript II (Invitrogen) with random primers. RT-qPCR was performed using a LightCycler 96 system (Roche, Basel, Switzerland) with TB Green Premix Ex Taq II (Takara). Gene expression levels were calculated using the standard curve method and normalized to those of the ribosomal protein gene *Rplp0*. The primers used for RT-qPCR analysis are provided in Table S2.

#### Correlative microscopy and block-face imaging (CoMBI)

Three-dimensional reconstructed images of uteri were created by a CoMBI system (https://combi-3d.github.io/manual/) [41]. Briefly, fixed uteri were stained with PBS containing 1% tannic acid overnight, immersed in PBS containing 30% sucrose, and frozen. The tissue blocks were sliced at a thickness of 5 µm using a Leica CM3050S cryostat (Leica Microsystems K.K., Tokyo, Japan) at -20°C. Serial block-face images were captured using a digital camera (Sony a7RIII, Tokyo, Japan), a macro lens (Sigma APO MACRO 180 mm F2.8 EX DG OS HSM, Tokyo, Japan), and a teleconverter (Kenko HD pro 2x, Tokyo, Japan). Camera shutter release was regulated with CoMBI devices composed of a magnetic sensor and microcontroller. Images were processed using ImageJ, ilastik [67], and 3D Slicer [68]. A few sections were also collected and correlated with the block-face images to identify structures.

#### ELISAs of serum hormone levels

Blood samples were collected from females on day 4 of pregnancy, centrifuged (5000 × *g* for 10 min at 4 °C) to collect serum, and stored at −80 °C. The serum concentrations of 17β-estradiol and progesterone were estimated using an estradiol ELISA kit (cat# 501890; Cayman, Ann Arbor, MI) and a progesterone ELISA kit (cat# 582601, Cayman), respectively.

#### Rescue experiment of implantation failure by recombinant leukemia inhibitory factor (LIF) administration

Pregnant *Kmt2d*^*d/d*^ females were intraperitoneally injected with 10 μg of recombinant mouse LIF (BioLegend, San Diego, CA) at 10:00 and 18:00 on day 4 of pregnancy, as described previously [42]. Implantation sites were evaluated at 18:00 on day 6 of pregnancy. The efficiency of recombinant LIF was verified using a delayed implantation model mice, as we previously reported with minor modifications [4]. Briefly, delayed implantation was induced by ovariectomizing pregnant C57BL/6J females at 17:00 on day 3 of pregnancy and was maintained by daily subcutaneous injection of progesterone (1 mg per mice). Two days after ovariectomy, recombinant LIF was intraperitoneally administered to induce implantation. Implantation sites were evaluated 2 days after LIF administration.

#### Library preparation for RNA sequencing (RNA-seq)

Total RNA was isolated from uteri on day 4 using TRIzol reagent and purified using an RNeasy Mini Kit (Qiagen Inc., Hilden, Germany) after treatment with DNase. mRNA was purified with Oligo dT beads (NEBNext Poly (A) mRNA magnet Isolation Module; New England Biolabs (NEB), Ipswich, MA), and cDNA libraries were produced with a NEBNext Ultra II RNA Library Prep Kit (NEB) and NEBNext Multiplex Oligos (NEB) as described previously [69]. The quality and concentration of libraries were confirmed using an Agilent 2200 TapeStation (D1000; Agilent, Santa Clara, CA). Libraries combined at equal molecular amounts were analyzed on an Illumina Nova-seq6000 DNA sequencer with a 50 bp paired-end cycle sequencing kit (Illumina Inc., San Diego, CA).

#### Analysis of RNA-seq data

In RNA-seq analysis of murine endometrial samples, sequence reads were aligned to the mouse genome mm39/GRCm39 using HISAT2 version 2.2.1 with the default option [70]. The number of reads at each gene was counted using the featureCounts function in the Rsubread package (v2.10.5) [71]. Differential expression analysis was performed using the TCC R package (https://www.R-project.org/) version 1.30.0 [72]. Genes with a q-value < 0.05 were defined as differentially expressed genes (DEGs). A volcano plot was generated using R with the EnhancedVolcano package (v1.14.0). Gene ontology analysis was performed using ShinyGO 0.77 [73] with DEGs as input.

RNA-seq analysis of the human endometrium was performed as described previously [20]. Raw reads (accession number: GSE207362) were aligned to the human genome sequence GRCh38/hg38 using HISAT2 version 2.1.0, and reads per kilobase of transcript per million mapped reads (RPKM) values were determined using the featureCounts function in the Rsubread package.

### Statistical analysis

The two-tailed Mann–Whitney U test was used to compare data between two groups unless otherwise specified. *P*-values less than 0.05 were considered statistically significant. Data are presented as means ± standard deviation. All experiments were performed at least three times to validate the results. No statistical method was used to predetermine sample size as effect sizes were unknown.

### Supplementary information

movieS1_blockface

movieS2_VR

Supplemental Material

Dataset S1

## Data Availability

The next-generation sequencing data of murine uteri in this study have been deposited in the DNA Data Bank of Japan (DDBJ) Sequence Read Archive under the accession code DRA017380.
