# Supplementary material for: The histone methyltransferase KMT2D is essential for embryo implantation via regulating precise differentiation of endometrial cells
Source: Cell Death Discov. 2024 Aug 8;10:357. doi: 10.1038/s41420-024-02134-9 (PMC11310208; doi:10.1038/s41420-024-02134-9)
Supplement: Supplementary file 3 — Supplemental Material [file 41420_2024_2134_MOESM3_ESM.docx]

**Supplementary Files for**

**The histone methyltransferase KMT2D is essential for embryo implantation via regulating precise differentiation of the endometrial cells in mice.**

Ryosuke Kobayashi^1^, Yuki Tajika^2, 3^, Junki Kohmaru^1^, Sumiyo Morita^1^, Takuro Horii^1^, Yoichi Mizukami^4^, Shizu Aikawa^5^, Yasushi Hirota^5^, Izuho Hatada^1, 6^

**Affiliations**

1. Laboratory of Genome Science, Biosignal Genome Resource Center, Institute for Molecular and Cellular Regulation, Gunma University, Gunma, 371-8512, Japan.
2. Department of Anatomy, Gunma University Graduate School of Medicine, Maebashi, 371-8511, Japan.
3. Gunma Prefectural College of Health Sciences, Maebashi, 371-0052, Japan
4. Institute of Gene Research, Science Research Center, Yamaguchi University, Yamaguchi, 755-8505, Japan.
5. Department of Obstetrics and Gynecology, Graduate School of Medicine, The University of Tokyo, Tokyo 113-8655, Japan
6. Viral Vector Core, Gunma University Initiative for Advanced Research (GIAR), Gunma, 371-8511, Japan.

*Corresponding author:

Izuho Hatada

Laboratory of Genome Science, Biosignal Genome Resource Center, Institute for Molecular and Cellular Regulation, Gunma University, Gunma, 371-8512, Japan.
Tel: +81-27-220-8057

E-mail: [hatada@gunma-u.ac.jp](mailto:hatada@gunma-u.ac.jp)

**This Supplementary File includes:**

Tables S1–S3

Figures S1–S6

Legends for Figures S1–S6, Dataset S1, and Movies S1 and S2

**Other supplementary materials for this manuscript include the following:**

Dataset S1

Movies S1 and S2

| **Table S1**. Sequences of crRNAs and the knock-in donors used in this study | |
| --- | --- |
|  |  |
| **crRNA target sequence (20 mer + PAM)** | |
| Gene target (name of crRNA) | Sequence (5'-3') |
| Kmt2c intron 8 (Kmt2cL1) | GCAGTTGTAGTTCCCCCTAAagg |
| Kmt2c intron 13 (Kmt2cR1) | CGTGTGTAGGTAATAATCAGagg |
| Kmt2d intron 15 (Kmt2dL1) | CGAAGAGCTTACACGGCTGAggg |
| Kmt2d intron 19 (Kmt2dR1) | GAGTCCGTTAGGTGAACCTGtgg |
| The PAM is written in lower case. | |
|  |  |
| **The donor ssODN sequence used for development of *Kmt2c*- and *Kmt2d*-floxed mice** | |
| Name of ssODN | Sequence (5'-3') |
| Kmt2cL1loxP_AS_PS | tg*t*a*aatgagctaaatacctaataaaaaatggaaaaaaaaaccctaatactcatcctttaATAACTTCGTATAGCATACATTATACGAAGTTATggatccgggggaactacaactgctagtgatgccgtgttggcagggtgaactggaaagacaact*g*c*a |
| Kmt2cR1loxP_AS_PS | gc*a*g*ctgtgacactgcttctggcttctgataggatttctgccacactagggatccctctgATAACTTCGTATAGCATACATTATACGAAGTTATgaattcattattacctacacacgttaacctagccaagacacggcctggattataatacttgtt*t*a*t |
| Kmt2dL1_ASPSNew | tg*g*t*ggagaacaggagatgcctcagctgtgtcggggagggattcgaagagcttacacggcATAACTTCGTATAGCATACATTATACGAAGTTATaccggttgagggcagacctctgagcagccggccagccgggtttggacagagcactcttaacac*c*a*a |
| Kmt2dR1loxP_AS_PS | gg*g*a*ttctaagatgagtgctgttgcagaagcctgctatgtccagaacctgttaaccacagATAACTTCGTATAGCATACATTATACGAAGTTATaagcttgttcacctaacggactctctagctgccaagagacaggaagaactctacttgctgaag*g*c*c |
| The LoxP sequence is written in upper case. Asterisks indicate a phosphotioate bond. | |

| **Table S2.** Primers used for genotyping PCR | | |
| --- | --- | --- |
|  |  |  |
| Gene | Primer name | Primer sequence (5'-3') |
| *Kmt2c* | Kmt2c-P1 | AAGGTCTGACATTGGGCTTG |
|  | Kmt2c-P2 | GGAGGAGGTGGTACAGCAAA |
|  | Kmt2c-P3 | GTGGGCCTTTTAAGAACACG |
| *Kmt2d* | Kmt2d-P1 | AACCTGAGGGAAACGAACCT |
|  | Kmt2d-P2 | ACCTTGGTGATCTGGTGGAG |
|  | Kmt2d-P3 | GCAGGCCAATGAAGCTCTAC |

| **Table S3**. Primers used for quantitative PCR analysis. | | | |  |
| --- | --- | --- | --- | --- |
|  |  |  |  |  |
| Gene | Ref_seq | Forward primer | Reverse primer | Product size (bp) |
| *Kmt2d* | NM_001033276 | GGAAGTGCAAGTGGTGTGTG | TCCACATAGGGAGCATGACA | 142 |
| *Ltf* | NM_008522 | GGAAGCACGGTATTTGAGGA | CCAGGTGGCACTCCTTGTAT | 112 |
| *Muc1* | NM_013605 | GACATCTTTCCAACCCAGGA | GGGGTGACTTGCTCCTACAA | 200 |
| *Wnt4* | NM_009523 | CTGGAGAAGTGTGGCTGTGA | GGACTGTGAGAAGGCTACGC | 108 |
| *Wnt5a* | NM_009524 | AGACAGGCATCAAGGAATGC | GTCTCTCGGCTGCCTATTTG | 112 |
| *Areg* | NM_009704 | GAACCTGGAGGTGGTGACAT | TGTCATCCTCGCTGTGAGTC | 92 |
| *Hand2* | NM_010402 | CCGACGTGAAAGAGGAGAAG | TGGTTTTCTTGTCGTTGCTG | 81 |
| *Ihh* | NM_010544 | GAGCTCACCCCCAACTACAA | TGACAGAGATGGCCAGTGAG | 118 |
| *Il13ra2* | NM_007956 | TCTCTGGGCGACATTCTTCT | GCTTTGGTGTGAAGGGTCAT | 124 |
| *Foxa2* | NM_001291065 | AGCAGAGCCCCAACAAGA | AGAGAGAGTGGCGGATGGAG | 117 |
| *Lif* | NM_008501 | CCCCATTTGAGCATGAACTT | AGCAGCAGTAAGGGCACAAT | 117 |
| *Coch* | NM_001198835 | CTCGTTCAAGCCAGTGAACA | TACCCCCTCGGAAACCTACT | 106 |
| *Igfbp3* | NM_008343 | CAGGCAGCCTAAGCACCTAC | CTTTCCACACTCCCAGCATT | 91 |
| *Rplp0* | NM_007475 | AGATGCAGCAGATCCGCA | GTTCTTGCCCATCAGCACC | 59 |

Figure S1


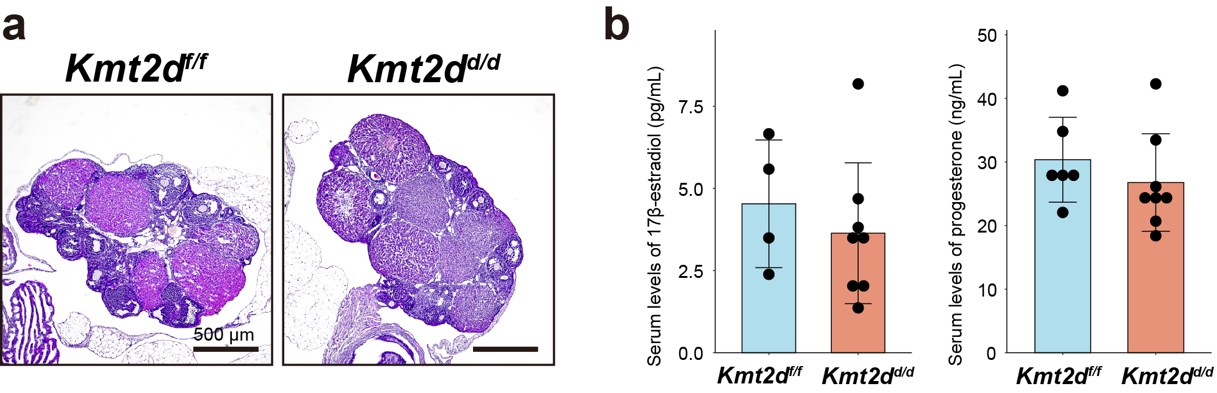


Figure S2


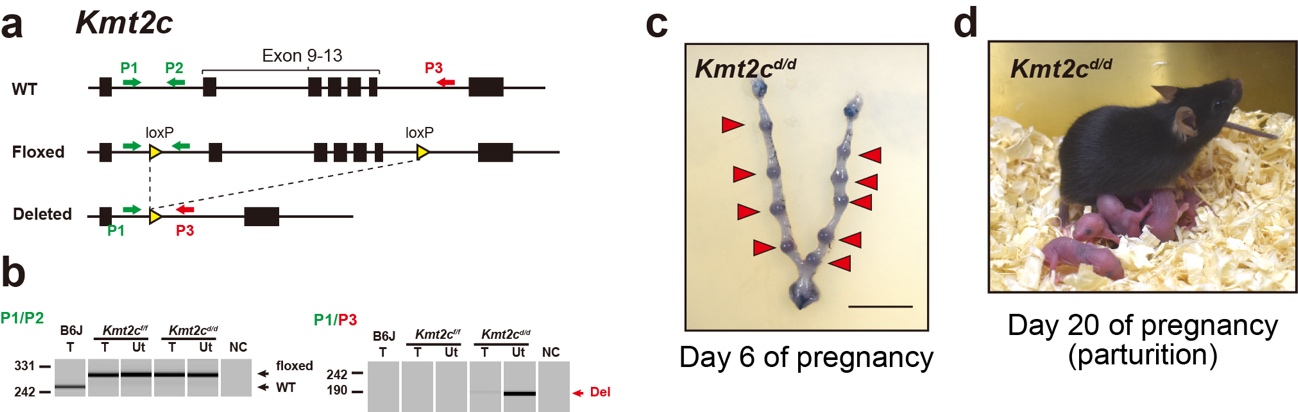


Figure S3


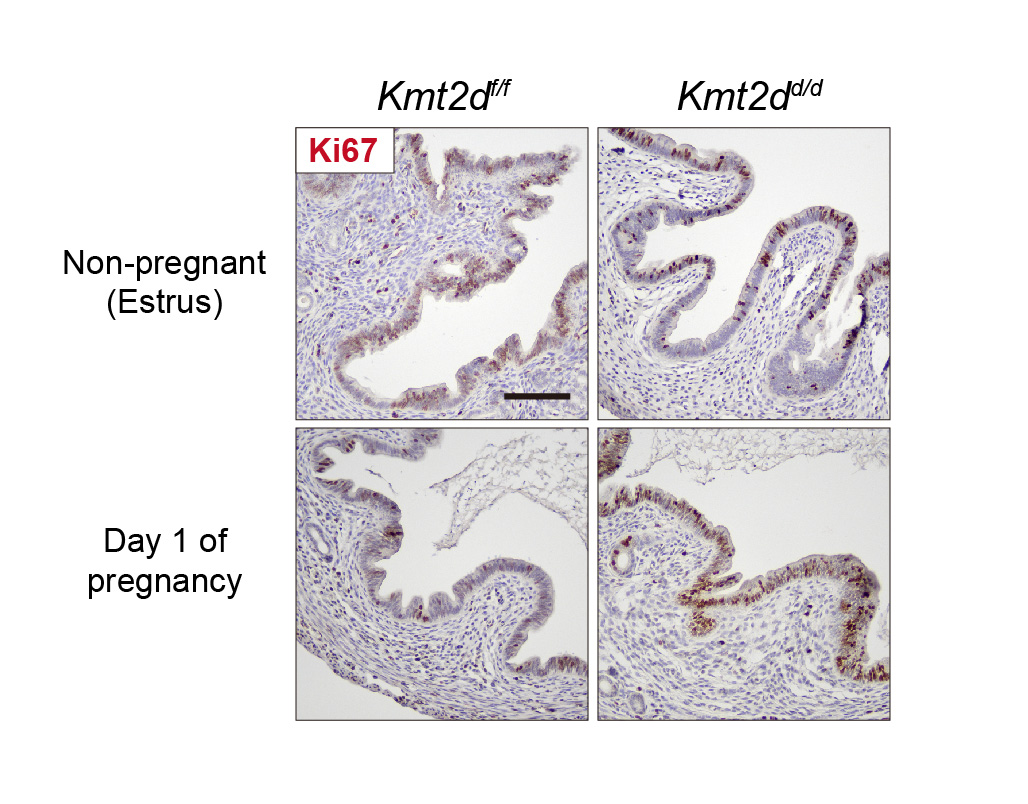


Figure S4

Figure S5.


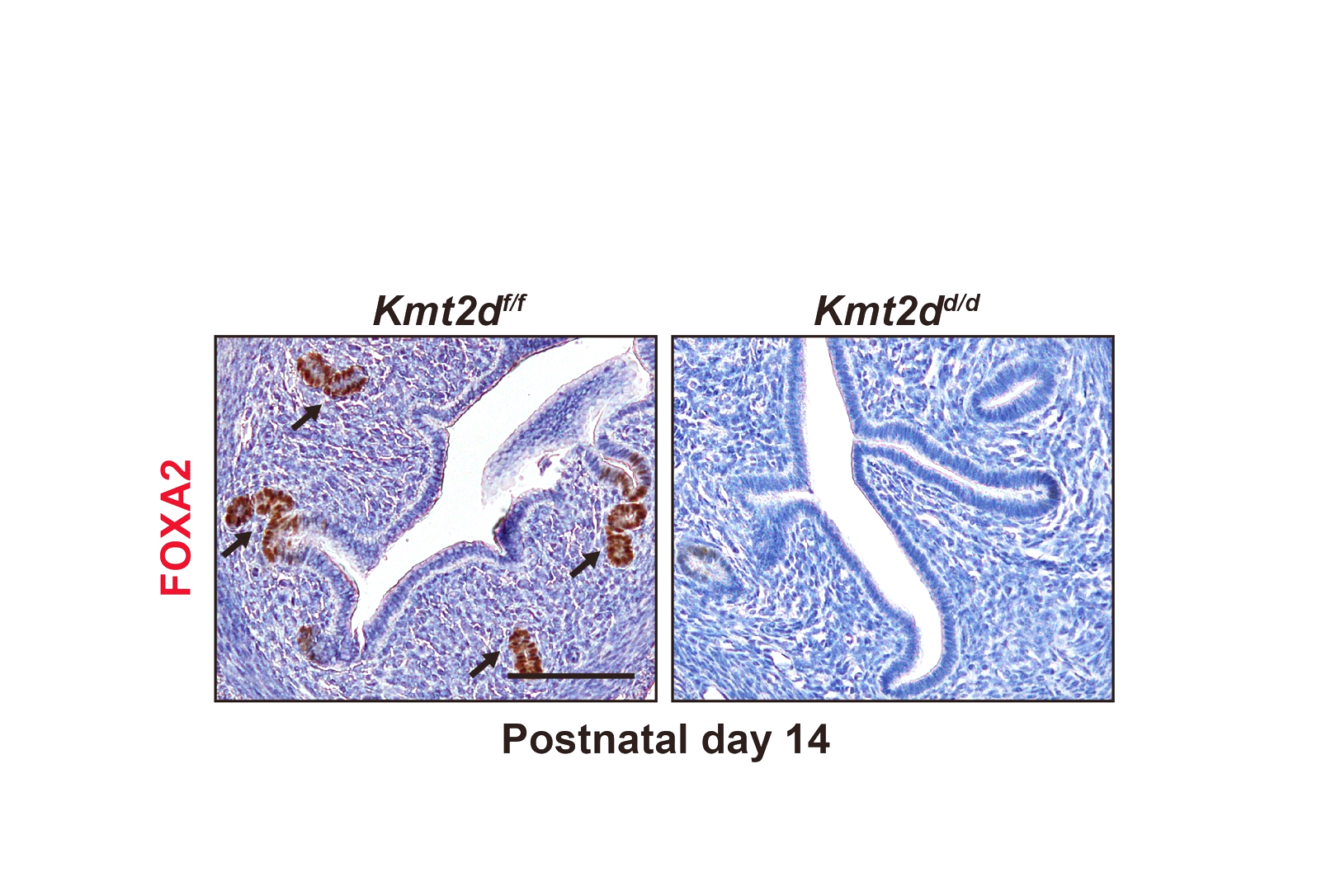


Figure S6


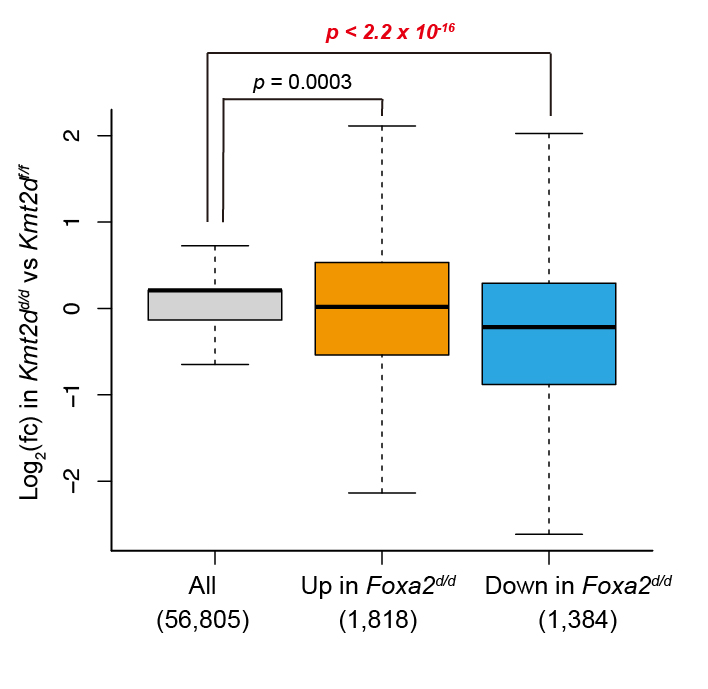


Figure S7


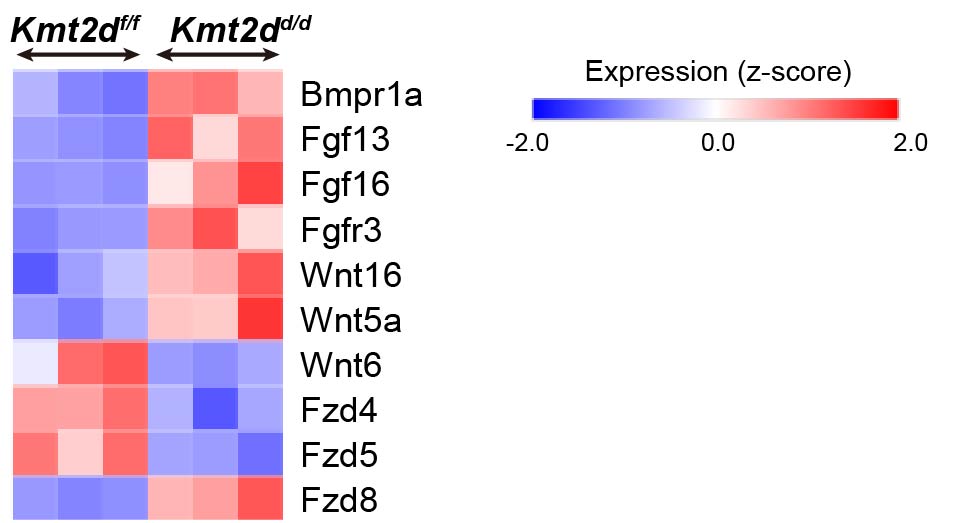


**Figure S1. Ovarian phenotypes in *Kmt2d^d/d^* mice.** (a) Representative images of the gross anatomy of the ovary. Scale bar = 500 μm. (b) Serum concentrations of E2 (left) and P4 (right) on day 4 of pregnancy. Levels of these hormones in *Kmt2d^d/d^* female mice were comparable with those in *Kmt2d^f/f^* females.

**Figure S2. Deletion of *Kmt2c* in the uterus has no effect on female fertility.** (a) The strategy to conditionally delete the *Kmt2c* gene. P1–3 indicate the locations of primers used for genotyping. (b) Genotyping PCR results showing *Kmt2c* was deleted in the uterus. T, tail DNA; Ut, uterus DNA; NC, negative control (water). (c) Representative image of the gross anatomy of uteri on day 5 of pregnancy in *Kmt2c^d/d^* mice. Implantation sites are indicated by red arrowheads. Scale bar = 10 mm. (d) Normal parturition was observed in a *Kmt2c^d/d^* female mouse on day 20 of pregnancy.

**Figure S3. Cell proliferation occurs normally in the uterine epithelium in *Kmt2d^d/d^* mice.** Ki67-positive epithelial cells were observed in both *Kmt2d^f/f^* and *Kmt2d^d/d^* uteri in estrus (non-pregnant) and on day 1 of pregnancy. Scale bar = 100 μm.

**Figure S4. 3D visualization of the uterus by CoMBI.** (a, b) Block-face images of control (a) and *Kmt2d^d/d^* (b) uteri viewed from the ovarian side. (c, d) Segmentation of uterine glands and the lumen was performed using ilastik and used for volume rendering. (e, f) Sections corresponding to block-faces (a, b) were stained with H&E and used to interpret block-face images. (g) The entire shape of the uterus was reconstructed from grayscale block-face images. Both control (upper image) and *Kmt2d^d/d^* (lower image) uteri were reconstructed using 1307 serial images each. (h) The uterine glands and lumen of control (upper image) and *Kmt2d^d/d^* (lower image) mice were segmented and shown by volume rendering. (i, j) The uterine glands and lumen in the region from planes (a and d) to 500 μm away viewed from the vaginal side. Scale bars = 500 μm. G: uterine gland, L: lumen.

**Figure S5. FOXA2-positive uterine gland cells are reduced in developing uteri.** Uteri on postnatal day 14 were immunostained for FOXA2. FOXA2 positive cells were indicated in black arrows. Scale bars = 100 μm.

**Figure S6. Downregulated genes in *Foxa2^d/d^* uteri during implantation are also repressed in *Kmt2d^d/d^* uteri.** Log2 values of fold changes in gene expression between *Kmt2d^d/d^* and *Kmt2d^f/f^* uteri were plotted. The gene set that was downregulated in *Foxa2^d/d^* uteri also showed significantly lower values compared with all genes (p < 2.2 × 10^-16^, two-tailed Mann-Whitney U test).

**Figure S7. DEGs involved in morphogen pathways in *Kmt2d^d/d^* uteri on day 4 of pregnancy.**  Expression levels of genes related in BMP, FGF, and WNT signaling are altered in *Kmt2d^d/d^* uteri.

**Dataset S1. List of differentially expressed genes in *Kmt2d^d/d^* uterus on day 4 of pregnancy compared to control.** The differentially expressed genes were defined by the threshold of q < 0.05 and more than 2-fold change.

**Movie S1. Blockface imaging of uteri and segmentation of uterine glands.** Uterus were sliced transversally and blockface images captured by CoMBI system. Segmentation of glands and lumen were performed using ilastik. The uterine glands are insufficiently developed in KO mouse.

**Movie S2. 3D reconstruction of uteri.** Serial blockface images (transversal planes) were used to create volume rendered images (uterine in blue-gray, glands in yellow-gray), and sagittal planes. All these images show insufficient development of glands in KO mouse.
